# Risk prediction score for death of traumatised and injured children

**DOI:** 10.1186/1471-2431-14-60

**Published:** 2014-02-28

**Authors:** Sakda Arj-ong Vallipakorn, Adisak Plitapolkarnpim, Paibul Suriyawongpaisal, Pimpa Techakamolsuk, Gary A Smith, Ammarin Thakkinstian

**Affiliations:** 1 Section for Clinical Epidemiology and Biostatistics, Faculty of Medicine, Ramathibodi Hospital, Mahidol University, Rama VI Road, Rajathevi, Bangkok 10400, Thailand; 2 Pediatric Ambulatory Units, Department of Pediatrics, Faculty of Medicine, Ramathibodi Hospital, Mahidol University, Bangkok 10400, Thailand; 3 Department of Community Medicine, Faculty of Medicine, Ramathibodi Hospital, Mahidol University, Bangkok 10400, Thailand; 4 Child Safety Promotion and Injury Prevention Research Center (CSIP), and Safe Kids Thailand, Bangkok 10400, Thailand; 5 Department of Disease Control, Ministry of Public Health, Nonthaburi, 11000 Thailand; 6 Center for Injury Research and Policy, Nationwide Children’s Hospital, Columbus, OH 43205, USA

**Keywords:** Logistic regression, Pediatric trauma and injury score, Prediction score, Injured child, Pediatric injury, Bootstrap

## Abstract

**Background:**

Injury prediction scores facilitate the development of clinical management protocols to decrease mortality. However, most of the previously developed scores are limited in scope and are non-specific for use in children. We aimed to develop and validate a risk prediction model of death for injured and Traumatised Thai children.

**Methods:**

Our cross-sectional study included 43,516 injured children from 34 emergency services. A risk prediction model was derived using a logistic regression analysis that included 15 predictors. Model performance was assessed using the concordance statistic (C-statistic) and the observed per expected (O/E) ratio. Internal validation of the model was performed using a 200-repetition bootstrap analysis.

**Results:**

Death occurred in 1.7% of the injured children (95% confidence interval [95% CI]: 1.57–1.82). Ten predictors (i.e., age, airway intervention, physical injury mechanism, three injured body regions, the Glasgow Coma Scale, and three vital signs) were significantly associated with death. The C-statistic and the O/E ratio were 0.938 (95% CI: 0.929–0.947) and 0.86 (95% CI: 0.70–1.02), respectively. The scoring scheme classified three risk stratifications with respective likelihood ratios of 1.26 (95% CI: 1.25–1.27), 2.45 (95% CI: 2.42–2.52), and 4.72 (95% CI: 4.57–4.88) for low, intermediate, and high risks of death. Internal validation showed good model performance (C-statistic = 0.938, 95% CI: 0.926–0.952) and a small calibration bias of 0.002 (95% CI: 0.0005–0.003).

**Conclusions:**

We developed a simplified Thai pediatric injury death prediction score with satisfactory calibrated and discriminative performance in emergency room settings.

## Background

On a global scale, injury is one of the most burdensome problems and the second most common cause of emergency department visits in children [1,2]. The mortality rate of injured children has decreased in developed countries, but the decrease has been slow and minimal in South East Asian developing countries. In Thailand, it has accounted for almost half of all causes of deaths since the 1990’s, and approximately 25% of deaths in children (overall average = 2.37–25.7/100,000 population) [3-6].

The Thai trauma care system was developed in the year 2000 to improve quality of care, reduce morbidity and mortality rates, and reduce the cost of injury treatment [7,8]. Factors associated with survival of injured children include individual characteristics (e.g., age, gender, weight, and underlying diseases), pre-hospital factors (e.g., injury mechanisms, anatomic injured regions, cause of injury, duration of transportation, and quality of first aid), and hospital factors (e.g., trauma center type, trauma care team experience, quality of emergency care, and the patient’s physiologic reserve at arrival). These factors were used to develop clinical prediction scores to predict injury severity and survival probability, and decrease the number of post-injury fatal outcomes. Emergency care personnel use these scores to prioritize proper treatment and management, allocate the trauma center type, physician, and team, and guide decisions about treatment interventions.

The Trauma Injury Severity Score (TRISS) [9-12] is the most well-known prediction score. It incorporates the Revised Trauma Score (RTS) [13] and the Injury Severity Score (ISS) [14]. However, the TRISS is adult-based and thus unsuitable for use in children [15-17]. The Pediatric Age Adjusted TRISS score (PAAT) [18] was developed by modifying the TRISS to be more specific for use in children. However, this score has some limitations because it has not been externally validated, does not use adjusted variable weighting, and only uses the three most severely injured body regions (out of a possible six), even though multiple regions may be injured. The New Injury Severity Score (NISS) [19-22] addresses this problem by summing the scores of the three most severe injuries regardless of body region, but does not account for the relative effect on outcome that injury of one body region may have compared with another. The Pediatric Trauma Score (PTS) [23,24] was designed to improve triage and management of injured children. Unfortunately, this score performs poorly for cases of blunt abdominal trauma, because it does not include body region. Given the poor performance of previously developed prediction scores, an alternative approach for score development was investigated by considering original variables individually rather than scoring them before including them in the equations. This approach accounts for the fact that different variables have different effects on survival. Logit model results were used to weight individual variables. We also considered for inclusion some variables (i.e., duration of transportation, type of injury, pre-hospital airway management) that are not included in the previously developed scores, but that may be relevant for our clinical setting.

The aim of this study was to develop and validate a simplified Thai pediatric trauma and injury prediction score of death. A scoring scheme and risk stratifications were created, and their performance was compared with the original [23] and modified PTSs [24-26].

## Methods

### Study design and setting

A multicenter cross-sectional study was performed during April 2010 to October 2012. The study was organized by the Thai Taskforce of Pediatric Injury, a collaboration between Ramathibodi Hospital (Bangkok), the Bureau of Epidemiology, the Ministry of Public Health (MOPH), and trauma care centers registered with the National Pediatric Injury and Trauma Registry of Thailand (NPIRT). Thirty-four trauma care centers (12 (47%), 11 (28%), and 11 (25%) hospitals representing trauma care levels I, II, and III–IV, respectively) participated in the study. The trauma care levels were classified based on the MOPH National Master Plan 1998–2009 [27].

### Selection of participants

Children aged 0–18 years who presented at the emergency services of collaborating hospitals with the following trauma or injury were included in the study: falling, being struck by or against, cut or pierce, gunshot wound, animal bite, transport injury, injury from child abuse, burn or scald, firearm-gun, foreign body aspiration, and drowning or near drowning. The study was approved by the Institutional Review Boards (IRBs) of the Faculty of Medicine Ramathibodi Hospital and the MOPH.

### Data collection and processing

Before the study was initiated, the research objectives and the roles of the collaborating sites were described to doctors and nurses that attended a collaborative meeting organized by our research team. Descriptions of pediatric injury and trauma, and the study variables and their measurements were standardized. The data were collected at the collaborative sites and were then transmitted to the central NPIRT database (http://nrpi.mahidol.ac.th), where all trauma cases were registered. The registration forms included patient demographic data, pre-hospital data, injury factors and their associated risks (type and mechanism of injury, site of injury, and injured body region), the Glasgow Coma Scale (GCS), vital signs, diagnosis-disposition, and outcome. Web-databases were constructed using PHP version 5.2.9 (PHP Group, Chittagong, Bangladesh) and MySQL client version 5.0.51a (Oracle Corporation, Redwood Shores, CA USA) software. Data were directly entered from individual trauma care centers in real-time. A quality control program for data entry was created based on possible values, variable codes, and cross-checks to verify and validate data. Data were checked by summarizing and cross-tabulating between relevant variables. The local collaborative sites were contacted when data were incorrect or missing, and the original medical records were consulted to determine the correct values.

### Variable and outcome measures

The outcome of interest was death related to injury or trauma within 30 days. The six domains of predictive variables were collected which were

–Demographic and general data including age, sex, weight, height, occupation, and geographic region.

–Pre-hospital data were transport types and duration, prior communication, and trauma care level.

–Mechanism of injury including surgical perspective mechanism (i.e., blunt, penetrating, or both) and physiological mechanism (i.e., gravity related injury, velocity related injury, or both).

–Trauma related injury regions including brain and head/neck, face, thorax, abdomen, upper or lower extremities and external soft tissue injury.

–Airway management which were intervention, airway adjuncts (e.g., oxygen supplementation and positive ambulatory bag, etc.)

–GCS and vital signs including GCS, Pulse rate (PR), systolic blood pressure (SBP) and respiratory rate (RR).

The route of transportation was sub-group based on modes of transportation in Thailand. Own transport defined as transported by the patient or their parent, non-ambulance group was transported by non-ambulance services or organized by a charity or a foundation supervised by EMTs or paramedics, and ambulance service was supervised by doctors, emergency physicians, and registered or emergency nurses.

Vital signs were measured at the emergency room and classified as follows [28]:

The SBP was defined as abnormal if SBP <60 for neonates, <70 for infants, <70 + (2 × age in years) for 1–10 years and <90 mmHg for >10 years. Otherwise it was classified as normal. PR was classified as tachycardia if PR >190 for ≤2 years, >140 for >2-10 years, and >100 beats/min for >10 years. Bradycardia was defined as PR < 60 beats/min. Pediatric Basic and Advanced Life Support criteria were used to classify RR as normal or tachypneic [29]. Consciousness consisted of awake, response to verbal stimulus, response to painful stimulus, and unresponsiveness. The original and the modified PTS were calculated using variables identified by Tepas et al. [23,25] and the modified Pediatric Polytrauma score 2012 [26].

### Primary data analysis

Mean and standard deviation (SD) were used to describe continuous variables if data were normal distribution, otherwise median and ranges were used. Frequency and percentage were used to describe categorical data. An overall death rate along with its 95% confidence interval (95% CI) was estimated. Data analysis consisted of 2 phases as follows;

### Derivation phase

The 21 independent variables were included in a data set that was used to develop risk prediction of death. A simple logistic regression analysis was used to evaluate the association between mortality and each of the variables. Variables with a p-value < 0.10 were included in a multivariate logistic model. The likelihood ratio (LR) test with backward elimination of variables was used to determine the most parsimonious model. Calibration and discrimination performance of the final model was then assessed. For calibration performance, a goodness of fit of the final model was assessed using the Hosmer-Lemeshow test [30]. A ratio of observed to expected values (O/E) was also estimated. A receiver operating characteristic curve (ROC) analysis was used to estimate discriminative performance, and the C-statistic was estimated.

The coefficients of the variables included in the final model were used to create scoring schemes. Total scores were calculated by summing the coefficients of all significant variables. The ROC analysis was applied to calibrate score cut-offs by estimating a likelihood ratio positive (LR^+^) for each distinct score cut-off. The prediction scores were then classified into risk stratification for ease of application in clinical practice [31].

### Validation phase

Because the death rate was quite low, all data were included in the 200-repetition bootstrap model used for internal validation. For each sample, the final logistic model resulting from the derivation phase was constructed, and parameters (i.e. predicted probability and the C-statistic) were estimated. Correlations between the observed and predicted values were assessed using the Somer’D correlation statistic (D_boot_). Model calibration was then assessed using D_orig_-D_boot_, where D_orig_ was the Somer’D correlation obtained from the derived data. A value close to 0 implied an optimistic calibration. Discrimination was also assessed by comparing the C-statistics results of the original model with the bootstrap modelling results [32-35].

Score performance was compared with the pre-existing PTSs using ROC curve analysis. Net reclassification improvement (NRI) and integrated discrimination improvement (IDI) statistics were also applied [36,37]. These measures allowed us to analyze benefit gains and losses when using our prediction scores compared with the PTSs scores. All analyses were performed using STATA 12.0 software (College Station, TX, USA) [38]. A P-value <0.05 was considered to be statistically significant.

## Results

### Characteristics of study subjects

The data from 43,561 injured children who presented at the emergency medical services of the 34 participating hospitals were entered and retrieved from the NPIRT databases during the study period. Of these, 13,382 (31%), 11,750 (30%), 7,529 (17%), 4,638 (11%), 3,430 (8%), and 2,832 (7%) injured children were from the north-eastern, southern, central, eastern, northern, and Bangkok areas of Thailand, respectively (Additional file 1: Table S1).

The mean age of the children was 11.4 ± 5.5 years, median weight was 45 kg (range = 7–76), and 71% were male (Table 1). Approximately 92% of them were injured while in their residential areas, and 39% were transferred to the hospital by ambulance. 47% had prior communication with the referral hospitals before transportation. Approximately 49% of the children received first aid at the trauma site scene, and 87% were provided appropriate assistance. Blunt injury (72%) was the most common mechanism of injury, followed by penetrating injury (14%). The three most common injuries were transportation (46%), falling (18%), and cut and pierce (8%) injuries.

**Table 1:** Descriptive characteristics of children

**Characteristics**	**N (%)**
Number of subjects	43,561
Demographic data	
Age, years, mean ± SD	11.37 + 5.52
Sex	
Male	30,883 (70.96)
Female	12,678 (29.10)
Weight, kg, median (min–max)	45 (7–76)
Occupation	
Parent care	7,513 (17.25)
Student	25,790 (59.25)
Other	10,258 (23.50)
Region	
Bangkok	2,832 (6.50)
Central	7,529 (17.28)
North	3,430 (7.87)
North-east	13,382 ( 30.72)
East	4,638 (10.65)
South	11,750 (26.97)
Injury location	
Resident province	40,210 (92.33)
Non-resident province	3,342 (7.67)
**Pre-hospital information**	
Transfer route	
Own transport	12,483 (28.66)
Non-ambulance	14,137 (32.45)
Ambulance	16,941 (38.89)
Prior communication	
Yes	23,120 (53.07)
No	20,441 (46.93)
Trauma level	
I	20,492 (47.04)
II	12,441 (28.56)
III	7,220 (17.57)
IV	3,408 (7.82)
Pre-hospital support	
Not needed	22,248 (51.07)
Not provided	2,812 (6.46)
Provided	18,498 (42.47)
**Injury types and mechanisms**	
Injury Mechanisms	
Blunt	31,482 (72.27)
Penetrating	5,940 (13.64)
Both	1,943 (4.46)
Non-classified	4,196 (9.63)
Physical mechanisms	
Velocity-related	1,528 (8.75)
Gravity-related	8,191 (19.44)
Both	22,003 (52.21)
Non-classified	10,368 (24.60)
Types of injury	
Transportation	19,928 (45.75)
Falling	7,902 (18.14)
Poisoning	461 (1.06)
Animal bite or sting	1,641 (3.77)
Struck by or against	3,426 (7.86)
Cut or pierce	3,502 (8.04)
Burn or scald	1,005 (2.31)
Fire gun or explosion	1,582 (3.63)
FB aspiration or suffocation	1,359 (3.12)
Drowning or submersion	355 (0.81)
Abuse, assault, or neglect	2,400 (5.51)
Object-related injury	
Chemical or food product	801 (1.84)
Home or office, work place	12,730 (29.22)
Sports equipment	639 (1.47)
Weapons	2,047 (4.70)
Transportation-related	20,317 (46.64)
Natural objects (animal)	3,362 (7.72)
Miscellaneous	3,664 (8.41)
Length of stay, days, median (min-max)	2 (0–63)
**Outcomes**	
Major outcome	
Survival	42,821 (98.30)
Death	740 (1.70)
**Short term disabilities**	
Outcome	
Major	1,862 (4.40)
Minor	3,624 (8.40)
None	37,334 (87.20)

The estimated overall death rate was 1.7% (95% CI: 1.57–1.82). The death rate was highest for children from the eastern region of Thailand (2.41%, 95% CI: 1.97–2.85), and lowest in Bangkok (0.78%, 95 CI: 0.45–1.10). Drowning was the highest cause of death (8.0%), followed by weapon, fire-gun, bomb-explosion, or firework injury (2.6%).

### Derivation phase

**Figure 1:**
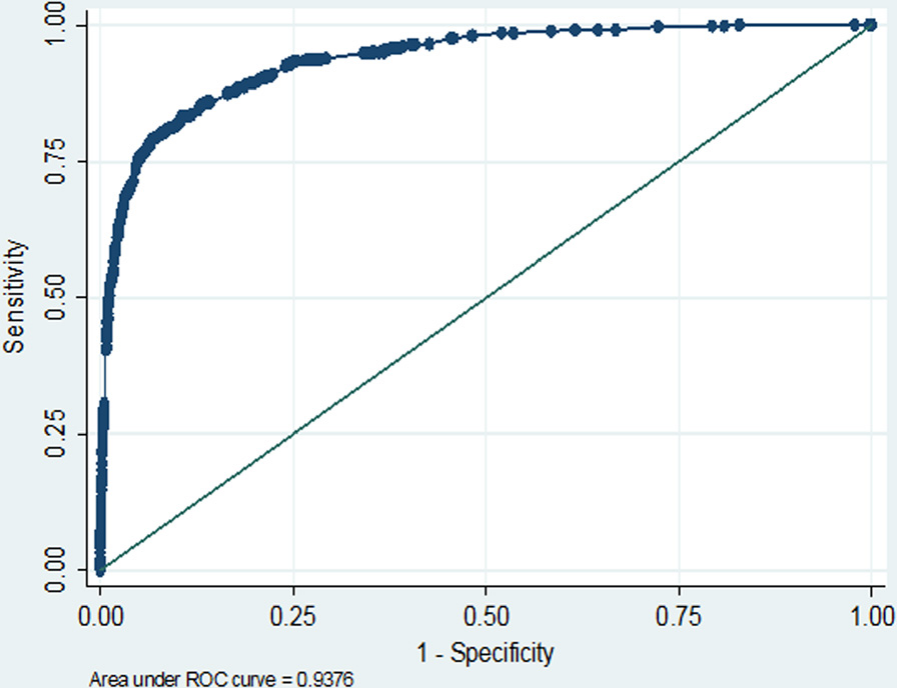
Receiver-operator characteristic (ROC) curve, Thai Pediatric Trauma and Injury Score, derivation data set.

The entire data set (n = 43,561 children) was used to derive the risk prediction score of death. The results of a univariate analysis revealed that 20 variables were significantly associated with risk of death (Table 2). Five variables exhibited multi-collinearity, so 15 variables were simultaneously included in the multivariate logistic model. Only 10 variables were significant and thus were retained in the final model (Table 3; logit equation presented in Additional file 1). The model displayed good fit to the data (Hosmer-Lemeshow Chi square = 13.64, d.f. = 5, p = 0.092; O/E ratio = 0.86, 95% CI: 0.70–1.02). The model was also effective at discriminating between dying and surviving children (C-statistic = 0.938, 95% CI: 0.929–0.947; read Figure 1).

**Table 2:** Factors associated with death, pediatric trauma and injury: univariate analysis

**Factors**	**Group**				
	**Death n (%)**	**Survival n (%)**	**OR**	**95% CI**	**P-value**
**Demographics domain**					
Age, years					
0–5	113 (1.2)	9,342 (98.8)	1.8	1.4–2.4	<0.001
6–12	131 (1.2)	10,478 (98.8)	3.01	2.1–4.3	
13–19	496 (2.1)	23,001 (97.9)			
Sex					
Female	175 (1.4)	12,503 (98.6)	1		0.001
Male	565 (1.8)	30,318 (98.2)	1.3	1.1–1.6	
Weight, kilograms					
≤25	147 (1.2)	12,335 (98.8)	1		<0.001
26– 45	134 (1.3)	9,909 (98.7)	1.1	0.9–1.4	
46–55	227 (2.1)	10,504 (97.8)	1.8	1.5–2.2	
>55	232 (2.3)	10,073 (97.8)	1.9	1.6–2.4	
**Pre-hospital domain**					
Duration of transport, hours					
≤ 1	142 (1.7)	8,312 (98.3)	1.2	0.9–1.5	
1–2	299 (2.0)	14,443 (98.0)	1.5	1.2–1.8	
2–3	138 (1.6)	8,555 (98.4)	1.2	0.9–1.5	
>3	161 (1.4)	11,511 (98.6)	1		0.001
**Airway management domain**					
No intervention	224 (0.6) 81	38,704 (99.4)	1		<0.001
Adjuncts	(3.9)	1,975 (96.1)	7.1	5.5–9.2	
Intubation	435 (16.9)	2,141 (83.1)	35.1	29.7–41.5	
**Mechanisms and injury regions domain**					
Velocity-, Gravity-related mechanism					
Velocity	41 (2.6)	1,541 (97.4)	3.0	2.0–4.3	<0.001
Gravity	97 (1.2)	8,160 (98.8)	1.3	1.0–1.8	
Both	510 (2.2)	22,844 (97.8)	2.5	2.0–3.1	
None	92 (0.9)	10,276 (99.1)	1		
Mechanism of injury					
Penetrating	69 (1.2)	5,871 (98.8)	1		<0.001
Blunt	536 (1.7)	30,946 (98.3)	1.5	1.1–1.9	
Both	56 (2.9)	1,887 (97.1)	2.5	1.8–3.6	
Non-classified	79 (1.9)	4,117 (98.1)	1.6	1.2–2.3	
No. of injured sites					
0	82 (2.2)	3,636 (97.8)	1		<0.001
1	168 (0.7)	25,519 (99.4)	0.3	0.2–0.4	
2	159 (1.8)	8,579 (98.2)	0.8	0.6–1.1	
≥3	331 (6.1)	5,087 (93.9)	2.9	2.3–3.7	
**Trauma body regions**					
Brain, head,neck					
Yes	527 (4.8)	10,355 (95.2)	7.8	6.6–9.1	<0.001
No	213 (0.6)	32,466 (99.4)	1		
Face					
Yes	43 (1.5)	2,903 (98.5)	0.8	0.6–1.2	0.298*
No	697 (1.7)	39,918 (98.3)	1		
Thorax					
Yes	124 (13.1)	821 (86.9)	10.3	8.4–12.6	<0.001
No	616 (1.4)	42,000 (98.5)	1		
Abdomen, pelvis					
Yes	143 (7.6)	1,743 (92.4)	5.6	4.7–6.8	<0.001
No	597 (1.4)	41,074 (98.6)	1		
Musculoskeletal					
Yes	161 (0.9)	18,045 (99.1)	0.4	0.3–0.5	<0.001
No	579 (2.3)	24,776 (97.7)	1		
External soft tissues					
Yes	171 (1.0)	16,196 (99.0)	0.5	0.4–0.6	< 0.001
No	569 (2.1)	39,918 (97.9)	1		
Wound types					
Large, open (major)	449 (2.7)	16,195 (97.3)	2.0	1.5–2.7	<0.001
Small, closed (minor)	247 (1.0)	23,466 (99.0)	0.8	0.5–1.0	
None	44 (1.4)	3,160 (98.6)	1		
Fracture types					
Open, multiple	115 (4.0)	2,756 (96.0)	2.5	2.0–3.0	<0.001
Single	198 (1.3)	14,962 (98.7)	0.8	0.7–0.9	
None	427 (1.7)	25,103 (98.3)	1		
**Severity domain**					
Total GCS In-Hospital					
<9	389 (18.6) 351	1,700 (81.4)	26.8	22.9–31.4	<0.001
≥9	(0.9)	41,121 (99.1)	1		
**Vital sign domain**					
PR					
Bradycardia	66 (15.1)	370 (84.9)	16.4	12.4–21.7	<0.001
Tachycardia	291 (3.8)	7,313 (96.2)	3.7	3.1–4.3	
Normal	383 (1.1)	35,138 (98.9)	1		
SBP					
Abnormal	164 (7.7)	1,960 (92.3)	5.9	5.0–7.1	<0.001
Normal	576 (1.4)	40,861 (98.6)	1		
RR					
Tachypnea	611 (2.1)	28,986 (97.9)	2.3	1.9–2.7	<0.001
Normal	129 (0.9)	13,835 (99.1)	1		
Consciousness (AVPU)					
Awake	313 (0.8)	39,722 (99.2)	1		<0.001
Verbal	33 (2.8)	1,161 (97.2)	3.6	2.5–5.2	
Pain stimulus	30 (7.4)	377 (92.6)	10.1	6.8–14.9	
Unresponsiveness	364 (19.0)	1,561 (81.1)	29.6	25.0–35.0	

**Table 3:** Results for multivariate logistic regression analysis of factors associated with the outcome variable, death

**Factors**	**Coefficient**	**SE**	**P-value**	**OR (95% CI)**
Age, years				
≤ 5	0.65	0.16	<0.001	1.9 (1.4–2.6)
6 – 12	1.09	0.19	<0.001	3.0 (2.0–4.3)
13 – 19				1
Airway management				
ET intubation	2.39	0.12	<0.001	10.9 (8.6–13.7)
Adjuncts	1.21	0.16	<0.001	3.3 (2.4–4.6)
None				1
Physical mechanism				
Velocity-related	0.24	0.14	0.08	1.3 (1.0–1.7)
Gravity-related	0.71	0.20	<0.001	2.0 (1.4–3.0)
Both	0.36	0.15	0.013	1.4 (1.1–1.9)
None				1
Head-neck injury				
Yes	1.61	0.10	<0.001	5.0 (4.1–6.1)
No				1
Thorax injury				
Yes	1.52	0.14	<0.001	4.6 (3.5–6.0)
No				1
Abdomen-pelvis injury				
Yes	1.62	0.13	<0.001	5.0 (4.0–6.5)
No				1
GCS				
<9	1.40	0.12	<0.001	4.0 (3.2–5.1)
≥9				1
PR				
Bradycardia	2.42	0.21	<0.001	11.3 (7.5–7.0)
Tachycardia	0.80	0.11	<0.001	2.2 (1.8–2.8)
Normal				1
SBP				
Abnormal	1.61	0.12	<0.001	5.0 (3.9–6.4)
Normal				1
RR				
Abnormal	0.79	0.21	<0.001	2.2 (1.5–3.1)
Normal				1

Magnitude of association was described using the odds ratio (OR) (Table 3). Children aged 1–5 and 6–12 years were at a 1.9 (95% CI: 1.4–2.6) and 3.0 (95% CI: 2.0–4.3) higher odds of death, respectively, than children aged 13–18 years. The odds of death for intubated children was about 10.9 (95% CI: 8.6–13.7) greater than the odds of death for non-intubated children. Children that received adjunct airway and support ventilation had a higher odds of death (OR = 3.3, 95% CI: 2.4–4.6) than children with non-airway support management.

The physical mechanism and region of injury domains were also significantly associated with death. Gravity, velocity, and both physical mechanisms were 2.0 (95% CI: 1.4–3.0), 1.3 (95% CI: 1.0–1.7), and 1.4 (95% CI: 1.1–1.9) times higher odds of death, respectively. Head (OR = 5.0, 95% CI: 4.1–6.1) and abdominal (OR = 5.0, 95% CI: 4.0–6.5) injuries were most strongly associated with the odds of death, followed by injury to the thorax (OR = 4.6, 95% CI: 3.5–6.0).

The odds of death for children with GCS < 9 was greater than the odds of death for children with a GCS ≥ 9 (OR = 4.0, 95% CI: 3.2–5.1). Abnormal PR, RR, and SBP were significantly associated with death. Children with bradycardia (OR = 11.3, 95% CI: 7.5–17.0) and tachycardia (OR = 2.2, 95% CI: 1.8–2.8) had a significantly higher odds of death than children with a normal PR. Compared with children with normal RR and SBP, children with an abnormal SBP and RR had a 5.0 (95% CI: 3.9–6.4) and 2.2 (95% CI: 1.5–3.1) times higher odds of death, respectively.

The total risk score (0–15.16) was created by summation of all coefficients for the variables that contributed to the final model (Table 4). For simplicity, and for easier application in clinical practice, the total risk score was classified into four stratifications according to its performance and distribution. The cut-offs were <1.02, ≥1.02, ≥1.96, and ≥3.06, which represented very low, low, intermediate, and high risks of death, respectively (Table 5). The LR^+^s for these corresponding risk stratifications were 1.26 (95% CI: 1.25–1.27), 2.47 (95% CI: 2.42–2.52), and 4.72 (95% CI: 4.57–4.88), respectively. The positive predictive values (PV^+^) for these four risk groups were 1.88% (95% CI: 1.74–2.04), 3.64% (95% CI: 3.36–3.94) and 6.73% (95% CI: 6.20–7.29), respectively.

**Table 4:** Thai pediatric trauma and injury scoring scheme

** Factors**	**Score**
1) Age, years	
≤ 5	0.65
6–12	1.09
≥13	0
2) Airway	
Intubation	2.39
Adjuncts	1.21
No intervention	0
3) Physical Mechanisms	
Pure velocity	0.24
Pure gravity	0.71
Both	0.36
None	0
4) Head-Neck injury	
Yes	1.61
No	0
5) Thoracic injury	
Yes	1.52
No	0
6) Abdomen-pelvis injury	
Yes	1.62
No	0
7) GCS	
<9	1.40
≥9	0
8) Pulse rate	
Bradycardia	2.42
Tachycardia	0.80
Normal	0
9) Respiratory rate	
Abnormal	0.79
Normal	0
10) Systolic blood pressure	
Abnormal	1.61
Normal	0
Total	0–15.16

**Table 5:** Risk classification of death, thai pediatric trauma and injury score

**Score cut-off**	**Risk groups**	**Score development discrimination capacities**					
		**Outcome**		**Sensitivity (%)**	**Specificity (%)**	**LR**^**+**^**(95% CI)**	**PV**^**+**^**(%)**
		**Death**	**Survival**				
<1.02	Very low	1	8,559				
≥1.02	Low	25	16,862	99.84	20.61	1.26 (1.25–1.27)	1.88 (1.74–2.04)
≥1.96	Intermediate	42	8,244	95.90	61.24	2.47 (2.42–2.52)	3.64 (3.36–3.94)
≥3.06	High	566	7,845	89.3	81.00	4.72 (4.57–4.88)	6.73 (6.20–7.29)

LR^+^, likelihood ratio positive; PV^+^, positive predictive value.

### Validation phase

The 200-replication bootstrap model yielded estimated D_boot_ and D_origin_ coefficients of 0.873 (95% CI: 0.872–0.875) and 0.872 (95% CI: 0.863–0.881), respectively, and a percentage error of 0.20%. The estimated bias was low, at 0.0017 (95% CI: 0.0005–0.0030), which indicated that the model was internally well-calibrated. The estimated O/E ratio for the bootstrap data were 0.86 (95% CI: 0.70–1.02), and the C-statistic was 0.938 (95% 95% CI: 0.926–0.952).

### Comparison of performances of prediction models

We compared our model to the original PTS developed by Tepas et al. [23,25] and the recently modified PTS, the Pediatric Polytrauma score 2012 [26]. The C-statistics for our score and the two other scores were 0.938 (95% CI: 0.929–0.947), 0.876 (95% CI: 0.862–0.891) and 0.874 (95% CI 0.860–0.888), respectively. Compared with the other two models, our model was significantly more likely to accurately discriminate between dying and surviving children (p < 0.001, Table 6).

**Table 6:** Comparison of model performance

**Models**	**ROC Area**	**95% CI**	**Survival RI**	**Death RI**	**NRI (95% CI)**
**Our model**	0.938	0.929–0.947	-	-	-
**Tepas (1987)**	0.876	0.862–0.891	+0.0564	+0.1057	+0.1621 (0.1122–0.2120)
**Pediatric Polytrauma (2012**)	0.874	0.860–0.888	−0.0294	+0.0442	+0.0148 (−0.0367–0.0663)

*NRI*, net reclassification improvement; *RI*, reclassification improvement.

The NRI was estimated by comparing our model to the two alternate models. The probability of death estimated from each model was classified using the previously estimated score cut-offs (Additional file 1: Tables S2 and S3). The reclassification tables were constructed by separately cross-tabulating the alternate model scores versus our scores by dying and surviving groups. Our model improved the classification of children in both the dying and surviving groups. The percent of reclassification improvements (RI) from the Tepas 1987 and the Pediatric Polytrauma score 2012 were 13.57% and 4.42% in the death group, with a loss of 2.9% and a gain of 5.6% in the survival group, respectively (Table 6). The NRIs were 16.2% (95% CI: 11.22–21.20) and 1.48% (95% CI: −3.67–6.63) for the Tepas 1987 and the Pediatric Polytrauma 2012 scores, respectively. This result indicated that compared with the Tepas 1987 model, the discrimination of our model was statistically superior. However, it was not an improvement on the Pediatric Polytrauma 2012 model.

## Discussion

Thirty-four hospitals across Thailand contributed data for a cross-sectional study of 43,561 injured and traumatized children. The most common injuries were transportation, falling, and cut and pierce injuries. Blunt injury was the most common mechanism of injury. The estimated overall death rate was 1.7%. The highest death rate occurred in the eastern region of Thailand (2.4%), and the lowest death rate occurred in Bangkok (0.78%). Drowning (8.0%) was the most common cause of death, followed by weapon, fire-gun, bomb-explosion, or firework injury (2.6%), and transportation (2.4%). The major causes of injured child death in Thailand were transportation (46%), falling (18%), and cut and pierce (8%) injuries. These results differ from results for the U.S., where transportation (48%), suffocation (19%), and drowning (13%) injuries represent the major causes of death for individuals 0–19 years in age [39]. In Europe, the major causes of death for individuals 0–19 years in age were transportation (23%), drowning (17%), and poisoning (7%) injuries [40].

The risk prediction score of death that was derived from our study indicated that 10 variables were significantly associated with death (age, intubation, physical mechanism, injury of head, abdomen, or thorax, GCS, PR, RR, and SBP). The derived model displayed a good fit to the data and discriminated dying from surviving subjects. The C-statistics were 0.938 (95% CI: 0.929–0.947), and 0.938 (95% CI: 0.926–0.952) for the derived and internally validated data, respectively. A simplified Thai pediatric trauma and injury scoring scheme was created, which indicated that children with a score >3 had a higher risk of death.

Emergency medicine has developed in Thailand since 2005, but the systems and services do not yet include all specialties, particularly specialties included in pediatric emergency medicine. Lack of human resources, medical equipment and supplies, low budgets, and lack of knowledge have contributed to this deficit. Most of the children that experience physical trauma and injury are treated by general or adult emergency physicians. A well-organized and maintained trauma/injury data registry for children still needs to be established, which would aid clinical decision-making for treatment management allocations. Our study should lead to the establishment of a data registry that includes the important variables necessary to create risk prediction models and severity grading systems. The risk prediction model should include user-friendly software to encourage health personnel in emergency settings to use it in routine practice.

### Ours versus previous risk scores

Few previous risk scores have been specifically developed for children (e.g., PTS [23-26], PAAT [18], NISS [19-22]). The PTS includes three variables that were included in our scoring system (i.e., airway, GCS, and SBP), but the other PTS variables were non-significant predictors in our model. Our model added seven significant variables (i.e., age, physical mechanism, three injured body regions (head-neck, thorax, and abdomen-pelvis), PR, and RR). Inclusion of individual body regions, and thus multiple injuries with different weights, was also considered based on the results of the logistic regression modeling. We also considered mechanism of injury, PR, and RR in our model. For PR, we considered bradycardia, tachycardia, and normal, which was more detail than simply using abnormal or normal PR. Therefore, our risk score was superior to the PTS [23] and to the other score, which are modified PTS score cut offs [24-26].

Although the NISS was specifically developed for children by modifying the ISS, it has not often been included in the TRISS [9-12]. This low use may be because NISS coding is complex, and the comprehensive detailed requirements of this system make it impractical for use as a triage tool.

### Risk factors for death

In our model, the association between airway management and death was similar to the PTS.

Children who were intubated had a risk of death 10 times greater than that of non-intubated children, and higher risk than other factors from multi-logit model (Table 3). The airway manipulation should be urgently performed to restore oxygenation and ventilation due to poor physiological reserve in children. These evidences were supported by Schafermeyer [41] which showed that aggressive airway and hemodynamic resuscitation were essential to critically injured child. Woosley et al. [42] emphasized that airway and ventilation were the first priority to improvement of thoracic injury in children. Likewise of severe traumatic brain injury, Boer et al. [43] showed the association of adequate airway management, prevention of hypoxia and hypo-hypercapnia were major components of trauma care improvement. Avarello et al. [44] and Brindis et al. [45] have also suggested aggressive resuscitation which included intubation was indicated to injured patient to improving their results.

Consciousness (measured by GCS) was also an important variable to predict death as an outcome. This result was similar to Cicero et al. [46] who found that only the GCS and Glasgow motor component could predict pre-hospital and on-arrival death. We considered vital signs by categorizing them as low, normal, and high, which was a more detailed approach than the abnormal and normal categories used by the PTS. As expected, for PR we found that bradycardia was associated with a greater odds of death than tachycardia (i.e., an approximately 11 times (bradycardia) and 2 times (tachycardia) higher risk of death than a normal PR). The effects of low values and high values were similar for the RR and SBP variables, so we combined them as abnormal RR and SBP. PR increased after the child was injured, which was an indication for early treatment administration and intervention. If management was delayed and the body could not continue to compensate, bradycardia would occur, blood pressure would drop, and shock would result.

Age was an important predictor of death. The odds of death were about 2 to 3 times higher for the children from the ≤5 and the 6–12 age groups than they were for the children from the 13–19 year age group. Only the PAAT has accounted for age effects via the Age-specific Pediatric Trauma Score (ASPTS). This age effect trend contrasted with Nance et al. [47], who found that compared with an older age of 13–15 years, a younger age had a protective effect. This difference might be explained by differences in exposure for the different age groups (i.e., dissimilar based line of physiologic reserve among age group, different types of trauma and injury result in differential injury severity and risk of death). Different countries also have different vehicle and road traffic safety regulations, which may indirectly affect trauma and injury risk in children.

Previous prediction scores included conventional mechanisms of injury (e.g., blunt and penetrating injuries), but these were not significant for our population. Physical mechanisms of injury (i.e., velocity and gravity) were significantly associated with death in our study and were included in our score. There was a greater odds of death for gravity-, compared with velocity-related injury.

The body regions head-neck, thorax, and abdominal-pelvis, were also important risk factors. These injured regions moderately affected the odds of death, with ORs of approximately 4.6–5.0. Our model considered injured regions individually and allowed the data from the logistic model to indicate which regions represented a significant risk, and how they should be weighted in the final score. Among six injured regions, only three of them were significant risk factors. The face, soft tissue, and musculoskeletal regions were not included. The weights of 1.61, 1.62, and 1.52 were applied to the head-neck, abdominal-pelvis, and thorax regions, respectively. Unlike other scores (e.g., AIS, ISS and TRISS), our score does not require additional calculations. This characteristic will reduce error at the trauma site scene.

### Calibration of scoring cutoff

The ROC curve analysis was used to estimate score cut-offs. The discrimination capability of each score was identified using LR^+^, which was a ratio of sensitivity versus 1-specificity. This parameter is useful for the selection of new diagnostic tests because it incorporates both sensitivity and specificity [48]. Unlike positive predictive value, LR^+^ does not depend on the prevalence/incidence of the event of interest. The LR^+^ indicates the degree to which a score cut-off would increase the pretest probability (or prevalence) of death. The User’s Guide for Evidence-based Medicine [49] specifies that LR^+^ Values of ≥10, 5–10, 2–5, and 1–2 should be respectively classified as conclusive, moderate, small but sometimes important, and very small changes in pretest probability of death. An examination of our results suggested that 3 cutoffs, ≥1.02, ≥1.96, and ≥3.06, with the respective LR^+^s of 1.26, 2.47, and 4.72, should be used. We designated these cutoffs as low, intermediate, and high risk of death, respectively. Children in the high risk group were approximately five times more likely to die than survive. Although none of our LR^+^ Values were as high as 10, they moderately shifted the pretest probability of death from 1.5% to 6.7%.

### Use of the Thai pediatric trauma and injury score

We encourage staff in emergency settings to use our score in routine practice among internally validated sites. Score estimation requires the measurement of 10 variables, and it is easily calculated (Table 4). The risk classification feature of our score should aid in the determination of whether patients should be transferred from, or treated at, a particular trauma care center, given the acute care facilities, equipment, and health care personnel. Only a patient with a low risk classification should be treated at a trauma care level III–IV hospital. A patient is classified as at intermediate risk classification may be treated (with close observation) at a level II hospital or transferred to a level I facility.

The outcomes will be compared and explored to find gap for improvement, and bring to develop the guidelines for trauma management of injured children in future. Within the scope of our study was developed injury prediction score of death for Thai injured children. This phase was only conducted among 34 multisite centers across Thailand with internal validation. We have not performed an external validation to ensure that the benefits of our score in different countries or networks have not been tested. The external validation is a next priority. A cross-sectional study that includes data from at least five provinces (one province for each region) will be collected using the same methods used in the score development phase. Development of portable personal computer software for score assessment is also necessary for widespread use of the score. Software development may be performed in parallel with the external validation phase or may be delayed until the results of external validation are complete. Before transfer to the user, the software should be tested for errors and for user satisfaction.

### Limitations

Some of the limitations indicated that the level of trauma care should be assessed and standardized. Most hospitals have been classified according to the size instead of available facilities. Improvements in transportation time will also improve the quality of trauma care in Thailand. A standardized trauma and injury transportation policy should be implemented. Development of the policy should include assessment of the availability of pediatric staff in emergency medicine, ambulance services for children, specialized medical instruments, and knowledgeable medical personnel. Consideration of these aspects will help policy makers to plan proper allocation of resources.

## Conclusions

A 10-variable risk prediction score of death was developed and validated. The variables included in the score were age, intubation, physical mechanism, head, abdomen, and thorax injury, GCS, PR, RR, and SBP. These variables are simple to assess and measure in routine practice. The scoring scheme is simple to calculate and interpret. Children with a high risk classification require prompt emergency treatment and management. Development of error-free and user-friendly software for installation in portable electronic is necessary so that widespread use of the score can be implemented.

## Abbreviations

AIS: Abbreviated injury scale; ASPTS: Age-specific pediatric trauma score; C-statistic: Concordance statistic; CI: Confidence interval; GCS: Glasgow coma scale score; IDI: Integrated discrimination improvement; IRBs: Institutional review boards; LR: Likelihood ratio; MOPH: Ministry of public health; MySQL: My-structured query language; NISS: New injury severity score; NPIRT: National pediatric injury and trauma registry of Thailand; NRI: Net reclassification improvement; O/E ratio: Observed per expected ratio; OR: Odds ratio; PAAT: Pediatric age adjusted TRISS score; PHP: Personal home page; PR: Pulse rate; PTS: Pediatric trauma score; PV+: Positive predictive value; RI: Reclassification improvements; ROC: Receiver operating characteristic curve; RR: Respiratory rate; RTS: Revised trauma score; SBP: Systolic blood pressure; TRISS: Trauma injury severity score.

## Competing interests

All authors declare that they have no competing interests.

## Authors’ contributions

SV, AP, GS, and AT contributed to the study concept and design. SV, PT, and AP were involved in the process of data acquisition. SV and AT performed data analysis, interpretation of results, and drafted the manuscript. SV and AT critically revised the manuscript. All authors participated in writing the pre-submission versions of the article and contributed substantially to its revision. All authors read and approved the final manuscript.

## Pre-publication history

The pre-publication history for this paper can be accessed here:

http://www.biomedcentral.com/1471-2431/14/60/prepub

## Supplementary Material

Additional file 1: Table S1 Collaborating hospitals by trauma care level (I–IV) and region. **Table S2.** Comparison with the Tepas 1987 model. **Table S3.** Comparison with the Pediatric Polytrauma Score 2012. S4. Logistic regression equation. Click here for file
