# Supplementary material for: Risk prediction score for death of traumatised and injured children
Source: BMC Pediatr. 2014 Feb 28;14:60. doi: 10.1186/1471-2431-14-60 (PMC3939810; doi:10.1186/1471-2431-14-60)
Supplement: Additional file 1: Table S1 — Collaborating hospitals by trauma care level (I–IV) and region. Table S2. Comparison with the Tepas 1987 model. Table S3. Comparison with the Pediatric Polytrauma Score 2012. S4. Logistic regression equation. [file 1471-2431-14-60-S1.docx]

**Supplements**

**Supplement Table 1.** Collaborating hospitals by trauma care level (I–IV) and region.

| **Trauma care level** | **No. of Hospitals** | **No. of studied hospitals** | **Region** | **Hospital** | **Children** |
| --- | --- | --- | --- | --- | --- |
| I | 44 | 12 | Central  North  North East  East  South | Ayuthaya  Saraburi l  Sawanpracharak  Chiangrai Regional  Maharat Nakornratsima  Khonkan  Sappasitthiprasong  Udonthani  Chonburi  Hatyai  Maharat Nakornsrithammaraj  Ratchaburi | 710  1,122  1,603  918  2,832  2,412  2,695  2,899  1,262  1,236  1,778  1,025 |
| II | 70 | 11 | Bangkok, Central  Central  North  North East  East  South | Ramathibodi  Nopparat & Lerdsin  Pranangklao Nonthaburi  Buddhachinaraj  Lampang  Surin  Chophayaabhaibhubejhr  Phrapokklao Chantaburi Suratthani  Yala | 2,354  478  356  1,052  1,283  2,414  626  828  1,974  1,076 |
| III–IV | 723 | 11 | Central  North  North East  East  South | Chaoprayayomraj Supanburi  Nakornpathom Uttaradit  Burirum Chachoengsao Rayong Chumphon  Vachira Phuket Krabi  Takuapa  Trang | 758  1,928  1,229  130  1,116  806  1,197  862  888  316  1,398 |
|  | 837 | 34 |  | Total | 43,561 |

**Supplement Table 2**. Comparison with the Tepas 1987 model.

| **Risk in Tepas**  **(Probability)** | **Risk in our model** | | | | | |
| --- | --- | --- | --- | --- | --- | --- |
| **Survival** | <0.0005 | 0.0005–0.0023 | 0.0023–0.0372 | 0.0372–0.7766 | >0.7766 | **Total** |
| <0.0005 | 12 | 756 | 637 | 20 | 0 | 1,425 |
| 0.0005–0.0023 | 62 | 10,182 | 7,242 | 523 | 1 | 18,010 |
| 0.0023–0.0372 | 34 | 11,104 | 7,809 | 973 | 0 | 19,920 |
| 0.0372–0.7766 | 0 | 351 | 986 | 803 | 10 | 2,150 |
| >0.7766 | 0 | 0 | 2 | 2 | 1 | 5 |
| Total | 108 | 22,393 | 16,676 | 2,321 | 12 | 41,510 |
| **Death** | <0.0005 | 0.0005–0.0023 | 0.0023–0.0372 | 0.0372–0.7766 | >0.7766 | **Total** |
| <0.0005 | 0 | 0 | 0 | 1 | 0 | 1 |
| 0.0005–0.0023 | 0 | 1 | 14 | 20 | 0 | 35 |
| 0.0023–0.0372 | 0 | 5 | 41 | 86 | 2 | 134 |
| 0.0372–0.7766 | 0 | 9 | 75 | 335 | 38 | 457 |
| >0.7766 | 0 | 0 | 1 | 4 | 2 | 7 |
| Total | 0 | 15 | 131 | 446 | 42 | 634 |

Reclassification index (RI) in surviving group:

Light gray color represents the number of injured children correctly reclassified by moving subjects down from high to low probability. Dark gray color represents the number of injured children incorrectly reclassified from our model by moving subjects up from low to high probability of death.

$$P\left( \mathrm{up} \right) = \frac{No. moving up}{N\left( \mathrm{survive} \right)}$$

$$= \frac{\left( 756+673+7242+20+523+973+1+10 \right)}{41510}$$

$$= 0.246$$

$$P\left( \mathrm{down} \right) = \frac{No. moving down}{N\left( \mathrm{survive} \right)}$$

= $\frac{(62+34+11104+351+986+2+2)}{41510}$

$= 0.302$ $\mathrm{RI}\left( \mathrm{survive} \right) = P\left( \mathrm{down} \right)-P\left( \mathrm{up} \right)$

$= 0.056$

Reclassification index (RI) in death group:

Light gray color represents the number of injured children correctly reclassified by moving subjects up from low to high probability. Dark gray color represents the number of injured children incorrectly reclassified from our model by moving subjects down from high to low probability of death.

$$P\left( \mathrm{up} \right) = \frac{No. moving up}{N\left( \mathrm{death} \right)}$$

$$= \frac{\left( 14+1+20+86+2+38 \right)}{634}$$

$$= 0.254$$

$$P\left( \mathrm{down} \right) = \frac{No. moving down}{N\left( \mathrm{death} \right)}$$

$$= \frac{\left( 5+9+75+1+4 \right)}{634}$$

$$= 0.148$$

$$\mathrm{RI}\left( \mathrm{death} \right) = P\left( \mathrm{up} \right)- P\left( \mathrm{down} \right)$$

$$= 0.106$$

Net Reclassification Improvement (NRI):

$$NRI = RI\left( \mathrm{death} \right) + RI\left( \mathrm{survive} \right)$$

$$= 0.106+0.056$$

$$= 0.162$$

**Supplement Table 3**. Comparison with the Pediatric Polytrauma Score 2012.

| **Risk in Pediatric Polytrauma Score**  **(Probability)** | **Risk in our model** | | | |
| --- | --- | --- | --- | --- |
| **Survival** | < 0.009 | 0.009–0.405 | > 0.405 | Total |
| < 0.009 | 30,713 | 4,774 | 18 | 35,505 |
| 0.009–0.405 | 3,546 | 2,270 | 73 | 5,889 |
| > 0.405 | 15 | 85 | 16 | 116 |
| Total | 34,274 | 7,129 | 107 | 41,510 |
| **Death** | < 0.009 | 0.009–0.405 | > 0.405 | Total |
| < 0.009 | 12 | 62 | 9 | 83 |
| 0.00 –0.405 | 61 | 295 | 81 | 437 |
| > 0.405 | 2 | 61 | 51 | 114 |
| Total | 75 | 418 | 141 | 634 |

Reclassification index (RI) in surviving group:

Light gray color represents the number of injured children correctly reclassified by moving subjects down from high to low probability. Dark gray color represents the number of injured children incorrectly reclassified from our model by moving subjects up from low to high probability of death.

$$P\left( \mathrm{up} \right) = \frac{No. moving up}{N\left( \mathrm{survive} \right)}$$

$$= \frac{\left( 4774+18+73 \right)}{41510}$$

$$= 0.117$$

$$P\left( \mathrm{down} \right) = \frac{No. moving down}{N\left( \mathrm{survive} \right)}$$

$$= \frac{\left( 3546+15+85 \right)}{41510}$$

$$= 0.088$$

$\mathrm{RI}\left( \mathrm{survive} \right) = P\left( \mathrm{down} \right)-P\left( \mathrm{up} \right)$

$= -0.0294$

Reclassification index (RI) in death group:

Light gray color represents the number of injured children correctly reclassified by moving subjects up from low to high probability. Dark gray color represents the number of injured children incorrectly reclassified from our model by moving subjects down from high to low probability of death.

$$P\left( \mathrm{up} \right) = \frac{No. moving up}{N\left( \mathrm{death} \right)}$$

$$= \frac{\left( 62+81+9 \right)}{634}$$

$$= 0.2397$$

$$P\left( \mathrm{down} \right) = \frac{No. moving down}{N\left( \mathrm{death} \right)}$$

$$= \frac{\left( 61+2+61 \right)}{634}$$

$= 0.19$56

$$\mathrm{RI}\left( \mathrm{death} \right) = P\left( \mathrm{up} \right)- P\left( \mathrm{down} \right)$$

$$= 0.0442$$

Net Reclassification Improvement (NRI):

$$NRI = RI\left( \mathrm{death} \right) + RI\left( \mathrm{survive} \right)$$

$= 0.0442-0.0294$

$$= 0.0148$$

**Supplement 4**. Logistic regression equation.

$$In\left[ \frac{P}{1-P} \right]=-7.82$$

$+ 0.65 x \left( Age\leq5 yrs \right)$
 $+ 1.09 x \left( Age 6-12 yrs \right)$

$$+ 1.21 x \left( Adjuncts Airway \right)$$

$$+ 2.39 x \left( Intubation \right)$$

$$+ 0.24 x \left( Pure velocity related injury \right)$$

$$+ 0.71 x \left( Puregravity related injury \right)$$

$$+ 0.36 x \left( Both velocity and Gravity related injury \right)$$

$$+ 1.61 x \left( Head-neck injury \right)$$

$$+ 1.52 x \left( Thoracic injury \right)$$

$$+ 1.62 x \left( Abdominal-Pelvis injury \right)$$

$$+ 1.40 x \left( GCS<9 \right)$$

$$+ 2.42 x \left( Bradycardia \right)$$

$$+ 0.80 x \left( Tachycardia \right)$$

$$+ 0.79 x (Dyspnea)$$

$$+ 1.61 x \left( Abnormal SBP \right)$$

*Abnormal vital signs: Systolic blood pressure (SBP), Pulse rate (PR; tachy-bradycardia), Respiratory rate (RR; Dyspnea) reference abnormality cut-off values from Pediatric Advanced Life Support (PALS), American Heart Association (AHA), 2010.*
